# Biomechanical evaluation of the hybrid pedicle screw—cortical bone trajectory technique in transforaminal lumbar interbody fusion to adjacent segment degeneration—finite element analysis

**DOI:** 10.1186/s12891-023-06411-z

**Published:** 2023-05-23

**Authors:** Rui Zhang, Alafate Kahaer, Hanqian Niu, Jingwen Wang, Ayididaer Jumahan, Yanning Qiu, Paerhati Rexiti, Hailong Guo

**Affiliations:** 1 Second Clinical Medical College, Xinjiang Medical University, Urumqi, China; 2 Department of Spine Surgery, The First Affiliated Hospital of Xinjiang Medical University, 137 Liyushan South Road, Urumqi, China; 3 Fifth Clinical Medical College, Xinjiang Medical University, Urumqi, China; 4 First Clinical Medical College, Xinjiang Medical University, Urumqi, China; 5 Xinjiang Key Laboratory of High Incidence Disease Research, Xinjiang Medical University, Ministry of Education, Urumqi, China; 6 Xinjiang Orthopedic Clinical Research Center, Urumqi, China

**Keywords:** Cortical bone trajectory screw, Pedicle screw, Lumbar spine, Transforaminal lumber interbody fusion, Finite element analysis

## Abstract

**Background:**

Transforaminal lumbar interbody fusion is an effective surgical treatment of intervertebral disk herniation. However, its clinical efficacy for adjacent segment disk degeneration (ASDD) after hybrid bilateral pedicle screw - bilateral cortical screw (pedicle screw at L4 and cortical bone trajectory screw at L5) and hybrid bilateral cortical screw - bilateral pedicle screw (bilateral cortical screw at L4 and bilateral pedicle screw at L5) remains undiscovered. Therefore, the aim of this study is to evaluate the effect of the hybrid bilateral pedicle screw - bilateral cortical screw and hybrid bilateral cortical screw - bilateral pedicle screw on the adjacent segment via a 3-dimensional (3D) finite element (FE) analysis.

**Methods:**

Four human cadaveric lumbar spine specimens were provided by the anatomy teaching and research department of Xinjiang Medical University. Four finite element models of L1-S1 lumbar spine segment were generated. For each of these, four lumbar transforaminal lumbar interbody fusion models at L4-L5 segment with the following instruments were created: hybrid bilateral pedicle screw - bilateral cortical screw, bilateral cortical screw - bilateral cortical screw (bilateral cortical screw at both L4 and L5 segments), bilateral pedicle screw - bilateral pedicle screw (bilateral pedicle screw at both L4 and L5 segments), and hybrid bilateral cortical screw - bilateral pedicle screw. A 400-N compressive load with 7.5 Nm moments was applied for the simulation of flexion, extension, lateral bending, and rotation. The range of motion of L3-L4 and L5-S1 segments and von Mises stress of the intervertebral disc at the adjacent segment were compared.

**Results:**

Hybrid bilateral pedicle screw - bilateral cortical screw has the lowest range of motion at L3-L4 segment in flexion, extension, and lateral bending, and the highest disc stress in all motions, while the range of motion at L5-S1 segment and disc stress was lower than bilateral pedicle screw - bilateral pedicle screw in flexion, extension, and lateral bending, and higher than bilateral cortical screw - bilateral cortical screw in all motions. The range of motion of hybrid bilateral cortical screw - bilateral pedicle screw at L3-L4 segment was lower than bilateral pedicle screw - bilateral pedicle screw and higher than bilateral cortical screw - bilateral cortical screw in flexion, extension, and lateral bending, and the range of motion at L5-S1 segment was higher than bilateral pedicle screw - bilateral pedicle screw in flexion, lateral bending, and axial rotation. The disc stress at L3-L4 segment was lowest and more dispersed in all motions, and the disc stress at L5-S1 segment was higher than bilateral pedicle screw - bilateral pedicle screw in lateral bending and axial rotation, but more dispersed.

**Conclusion:**

Hybrid bilateral cortical screw - bilateral pedicle screw decreases the impact on adjacent segments after spinal fusion, reduces the iatrogenic injury to the paravertebral tissues, and provides throughout decompression of the lateral recess.

## Introduction

Adjacent segment degeneration (ASD) after spinal fusion refers to abnormal pathological changes in the cephalic or caudal segments of the fused segment, including hypertrophic osteoarthritis, segmental instability, degenerative spondylolisthesis, and degeneration of the intervertebral disc. Transforaminal lumbar interbody fusion has been widely adopted for the treatment of various lumbar spine disorders to effectively correct deformities and restore stability of the lumbar spine. However, it has been documented that spinal fixation techniques increase the incidence of ASD, for the reason that while the stiffness of the fused segment was significantly increased, the functions it carries were compensated by the adjacent segment, which can cause overloads on the disc, ligaments, facet joints, and capsules [1]. Schulitz et al. [2] reported a 10% and 23% incidence of adjacent segment instability in postero-lateral lumbar fusions without and with internal fixation.

The risk of ASD after spinal fusion was related to patient’s age, BMI, severity of osteoporosis, decompression, and fusion approach, internal fixation, and postoperative stiffness of the fixed segment [1, 3]. Different fixation techniques have different effects on the adjacent segments. The damage to the facet joints during the pedicle screw implantation accelerates the degeneration of the adjacent segment [1]. And the occurrence of ASD reduces the long-term clinical outcome of the surgery and increases the revision rate [4]. In 2009, Santoni et al. [5] proposed the cortical bone trajectory screw (CBT) technique in which the insertion point was performed by moving inside to reduce the surgical incision, damage to muscles, soft tissues, and facet joints, shorten the operation time.

In transforaminal lumbar interbody fusion (TLIF), decompression of the lateral recess inferior to the fused segment was necessary [6]. Compared with the pedicle screw (PS) technique, the CBT technique may not provide throughout decompression because the scope for decompression was too close to the screw insertion point, which may affect the efficacy of the surgery to a certain extent [6]. To compensate for the deficiencies and to reduce the incidence of ASD, we previously proposed hybrid techniques with PS and CBT, and biomechanical effects on the fixation segment were discussed [6]. There was a paucity of literature on the biomechanics of hybrid fixation techniques with PS and CBT, most of which were cadaveric studies of anatomical specimens, and there were few studies on the effects of hybrid fixation techniques with PS and CBT on ASD in finite element method (FEM). In this study, we used finite element analysis to comparatively analyze the biomechanical effects of four fixation techniques on the cranial and caudal adjacent segments to provide guidance for cadaveric testing and clinical application of the combined screw placement technique.

## Materials and methods

### Model development of the L1-S1 lumber spine

**Fig. 1:**
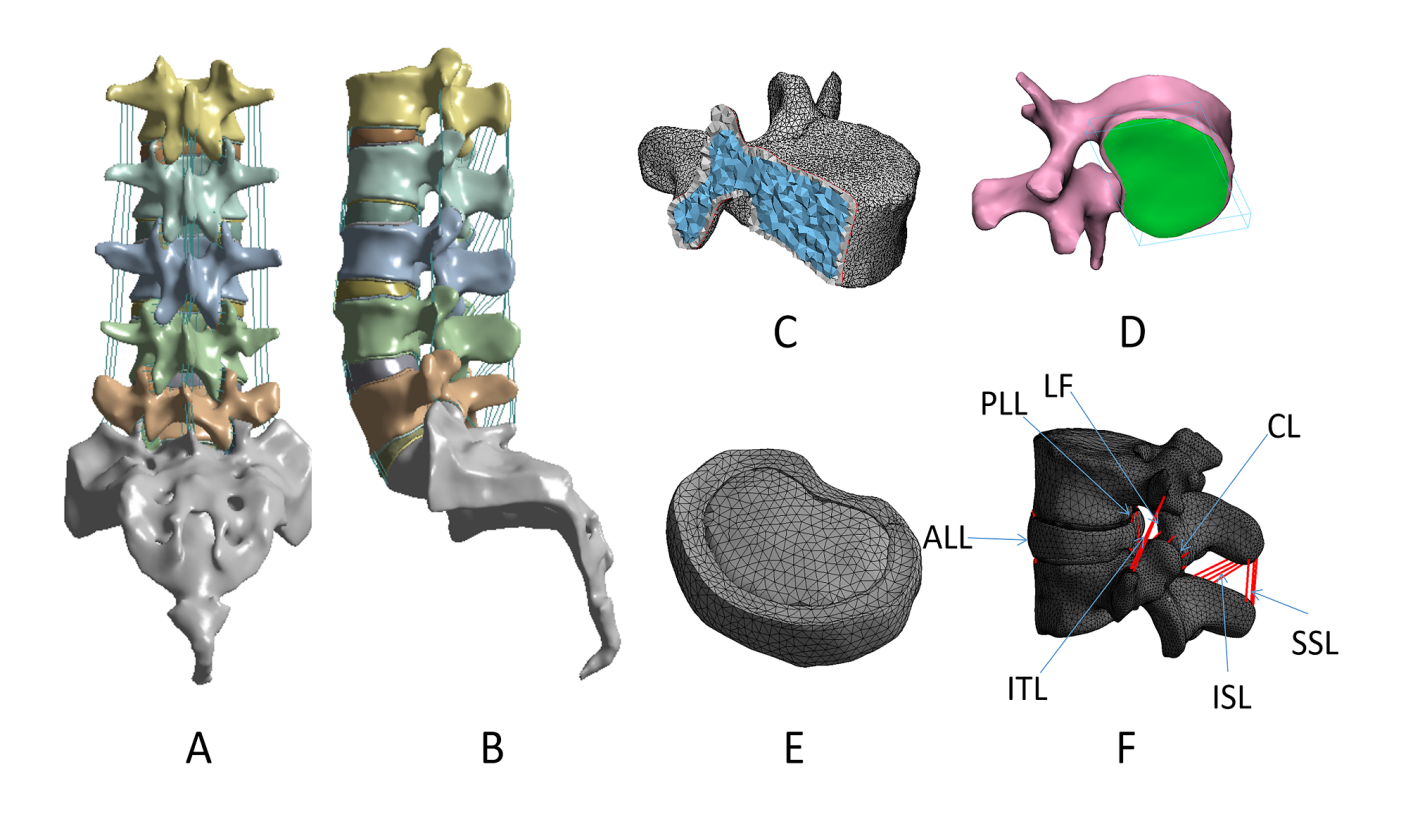
The finite element model of the intact L1-S1 lumbar spine. **(A)** Coronal view; **(B)** Sagittal view; **(C)** Regional thickness of the cortical bone; **(D)** Vertebral endplate; **(E)** Intervertebral disc; **(F)** Ligaments

Four human cadaveric lumbar spine specimens were provided by the anatomy teaching and research department of Xinjiang Medical University. Tumors, tuberculosis, and previous history of surgery were excluded. High-resolution computed tomography (AQUIRRON 16, PHILIPS, Netherlands) was performed on L1-S1 lumbar spine of the specimens, and the image data were saved in DICOM format. The original data were imported into Mimics 17.0 (Materialize, Leuven, Belgium) software, the lumbar spine segments were segmented layer by layer, the individual lumbar spine 3D coordinate system was re-established, the appropriate range of vertebral bodies was selected, and then the brush tool was used to edit the mask, fill in the gaps. The smooth 3D (three-dimensional) model was then saved in STL format and imported into 3-Matic software (Materialize, Leuven, Belgium) for further mesh division (Fig. 1A, B). The thickness of cortical bone and cartilage endplates was defined as 0.5-1 mm [7] and 1 mm [8](Fig. 1C, D). The nucleus pulposus was simulated as a fluid-like incompressible substance that occupied 44% of the disc volume [9] (Fig. 1E). The contact between the facet joint cartilages was defined as “soft frictionless contact” according to the natural distance on CT, with an initial gap of 0.5 mm [7]. The anterior longitudinal ligament (ALL), posterior longitudinal ligament (PLL), intertransverse ligament (ITL), ligamentum flavum (LF), capsular ligament (CL), interspinous ligament (ISL), and supraspinous ligament (SSL) were represented and assigned nonlinear material properties (Fig. 1F). The contact between the upper and lower ends of the disc and the surfaces of the vertebral body was defined as “bonding” contact [10]. A reference point was established at the center of the upper surface of the L1 vertebral body, which was coupled to the upper surface to apply compressive load and torque. Finally, the mesh division and material properties were set using ANSYS Workbench 19.1 (ANSYS, Inc., Canonsburg, PA, USA) [11–15] (Table 1**).** In the same case, the hexahedral mesh has higher accuracy and easier convergence than the tetrahedral mesh, but the lumbar spine model was an irregular structure and it was more difficult to perform hexahedral meshing, so in this study, tetrahedral elements were used for the cortical bone and cancellous bone, and hexahedral elements were used for the endplates, fibrous rings, and nucleus pulposus.

**Table 1:** Material properties in current study [11–15]

Materials	Young’s Modulus (Mpa)	Poisson’s Ratio	Density (g/cm3)	Cross Sectional Area (mm2)	Radius (mm)
Cortical bone	12,000	0.3	1.91		
Cancellous bone	100	0.2	1.87		
Cartilaginous endplate	23.8	0.4	1.0003		
Facet cartilage	24	0.4			
Annulus fibrosis	4.2	0.45			
Nucleus pulposus	1	0.4999			
ALL	7.8(< 12.0%) 20.0(> 12.0%)		1.00E-06	63.7	4.5029
PLL	10.0(< 11.0%) 20.0(> 11.0%)		1.00E-06	20	2.5231
CL	7.5(< 25.0%) 32.9(> 25.0%)		1.00E-06	30	3.0902
LF	15.0(< 6.2%) 19.5(> 6.2%)		1.00E-06	40	3.5682
ISL	10.0(< 14.0%) 11.6(> 14.0%)		1.00E-06	40	3.5682
SSL	8.0(< 20.0%) 15(> 20.0%)		1.00E-06	30	3.0902
ITL	10.0(< 18.0%) 58.7(> 18.0%)		1.00E-06	1.8	0.7569
Cage(PEEK)	3600	0.25	1.32e − 6		
Screw and Rod(Titanium)	110,000	0.3	4.5e − 6		

### Construction of surgical models

**Fig. 2:**
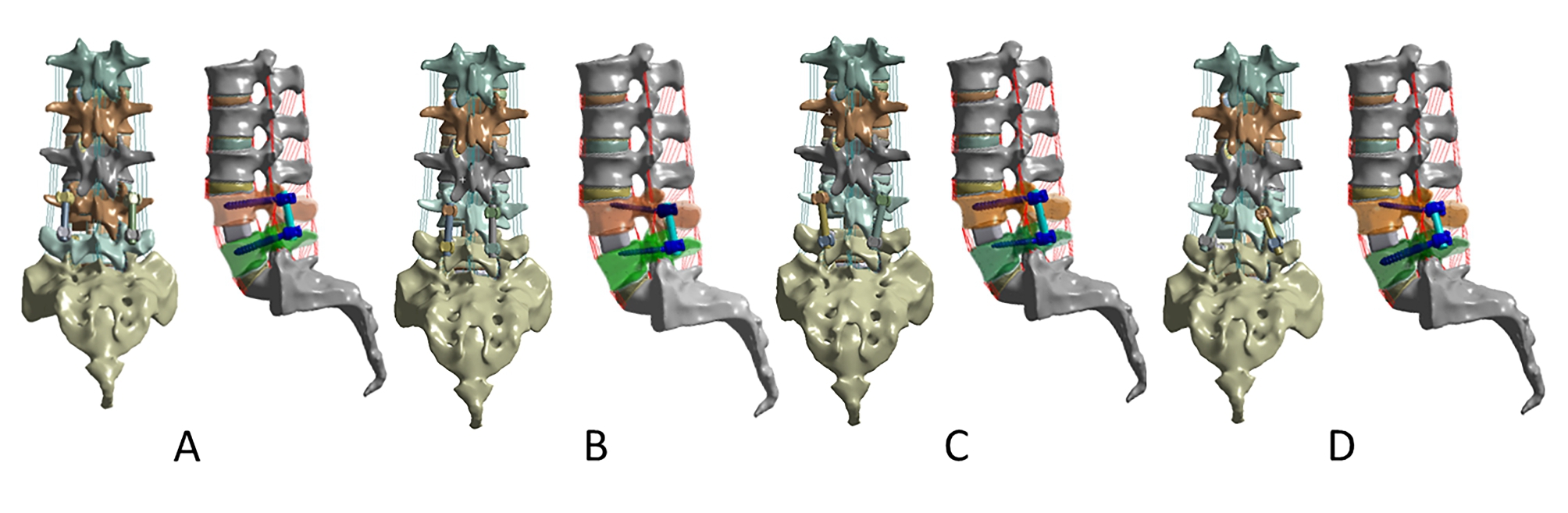
FE models of L1-S1 lumbar spine with TLIF at L4-L5 segment with four different fixation techniques. **(A)** PS at L4 and L5 (PS-PS); **(B)** CBT at L4 and L5 (CBT-CBT); **(C)** PS at L4 and CBT at L5 (PS-CBT); **(D)** CBT at L4 and PS at L5 (CBT-PS).

TLIF procedures were performed randomly on the right and left sides of L4-L5 segment in Mimics 17.0 (Materialize, Leuven, Belgium), followed by internal fixation according to the following techniques: (1) PS-PS (PS at both L4 and L5 segment) (Fig. 2A). (2) CBT-CBT (CBT at both L4 and L5 segment) (Fig. 2B). (3) PS-CBT (PS at L4 and CBT at L5) (Fig. 2C). (4) CBT-PS (CBT at L4 and PS at L5) (Fig. 2D). The diameter of the rod is 5.0 mm. Due to the different entry points and screw trajectories of the CBT and PS techniques, the diameter and length of the screw trajectories and the distribution of the surrounding bone are also different, and these differences result in different screw sizes for clinical application of the two techniques. The ideal specifications for CBT are: diameter greater than 5.5 mm and length greater than 35 mm [16]; the ideal specifications for PS are: diameter of 6 mm and length of 45 mm [17]. After analyzing the anatomical data of the selected specimens and referring to the studies of other scholars [18–23], we set the screw specifications as follows: PS with a diameter of 6.0 mm and length of 45 mm; CBT with a diameter of 5.0 mm and length of 35 mm.

### Model validation

**Fig. 3:**
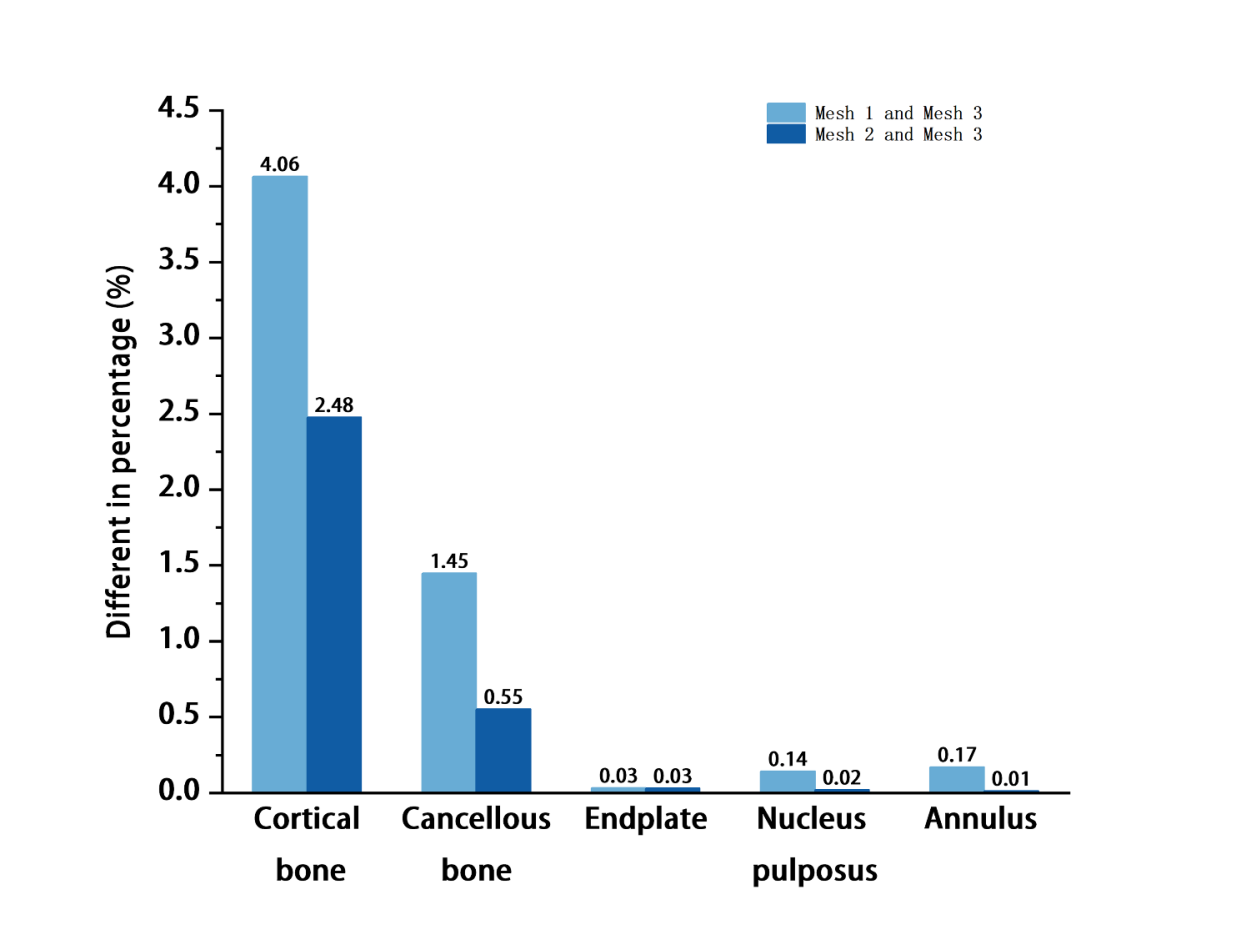
Predicted percentage differences of the von Mises stress between Mesh 1 and Mesh 3 and between Mesh 2 and Mesh 3 in a different component. The results show that the differences in von Mises stress between Mesh1 and Mesh3, Mesh2 and Mesh3 is less than 5%, and the difference between Mesh2 and Mesh3 is less than or equal to Mesh1 and Mesh3 in all aspects.

The validation of the intact FE model consists of two steps. First, mesh convergence was validated. Three different meshes were generated consecutively (mesh 1, mesh 2, and mesh 3). Among the three meshes, mesh 1 has the least number of elements and nodes, and mesh 3 has the most. Ayturk et al. [24] have shown that axial rotation was the most sensitive motion to the mesh resolution of the FE model, and the mesh was considered to converge when the difference between the predicted von Mises stresses of different components obtained by two successive mesh resolutions was less than 5% in rotation with a torque of 7.5 Nm. In this study, the differences between the von Mises stresses of the three meshes were compared by the identical method (Fig. 3). Second, 7.5 Nm moment and 400 N compressive load were applied to the model to simulate flexion, extension, lateral bending, and rotation, and ROM (range of motion) of the intact model in each segment was compared with that of Yamamoto et al. [25], Shim et al. [26], Huang et al. [27], Lo et al. [28].

### Boundary and loading conditions

The sacrum was completely fixed and restrained. 400 N compressive load and 7.5 Nm torque were applied to this reference point to simulate flexion, extension, lateral bending, and axial rotation, respectively. The ROM and peak von Mises stress of the intervertebral disc at L3-L4, and L5-S1 segments were recorded and analyzed.

### Statistical methods

SPSS 27.0 software was used for statistical analysis. The data was expressed as mean ± standard deviation. Paired t-test was used for the analysis of variance. When differences were statistically significant, post hoc test was performed using the least significant difference (LSD) method. All results were considered significant at P < 0.05.

## Results

### Model validation

**Fig. 4:**
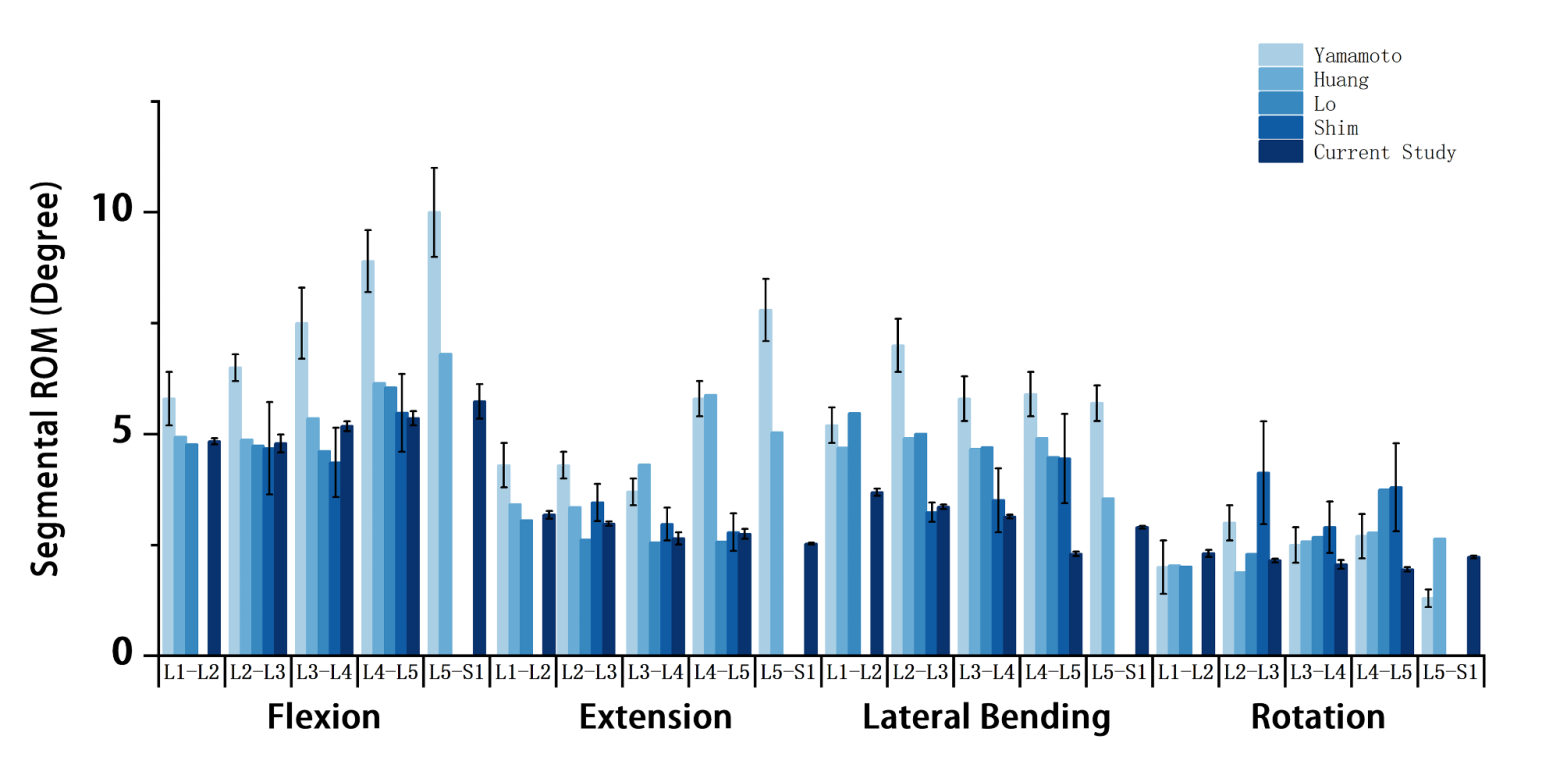
Comparison of ROM of each segment between the current intact FE model and the previous studies. We used the intact model developed in this study to simulate the movements of forward flexion, back extension, lateral bending, and axial rotation and recorded the activity of each segment, contrary to the results of Yamamoto et al., Shim et al., Huang et al., Lo et al. In this study, the degree of intersegmental activity was mostly greater in the intact model than in the models of Yamamoto et al., Shim et al., Huang et al., Lo et al. Although there are data differences, they are more consistent with the trend of data variation.

The difference in von Mises stresses between mesh 2 and mesh 3 was less than 5% in all components of the model, and mesh 2 was considered to be converged. Compared with the ROM in the previous literature [25–28] (Fig. 4), the results confirm that the model can be used for further biomechanical analysis.

### ROM of L3-L4 segment

**Fig. 5:**
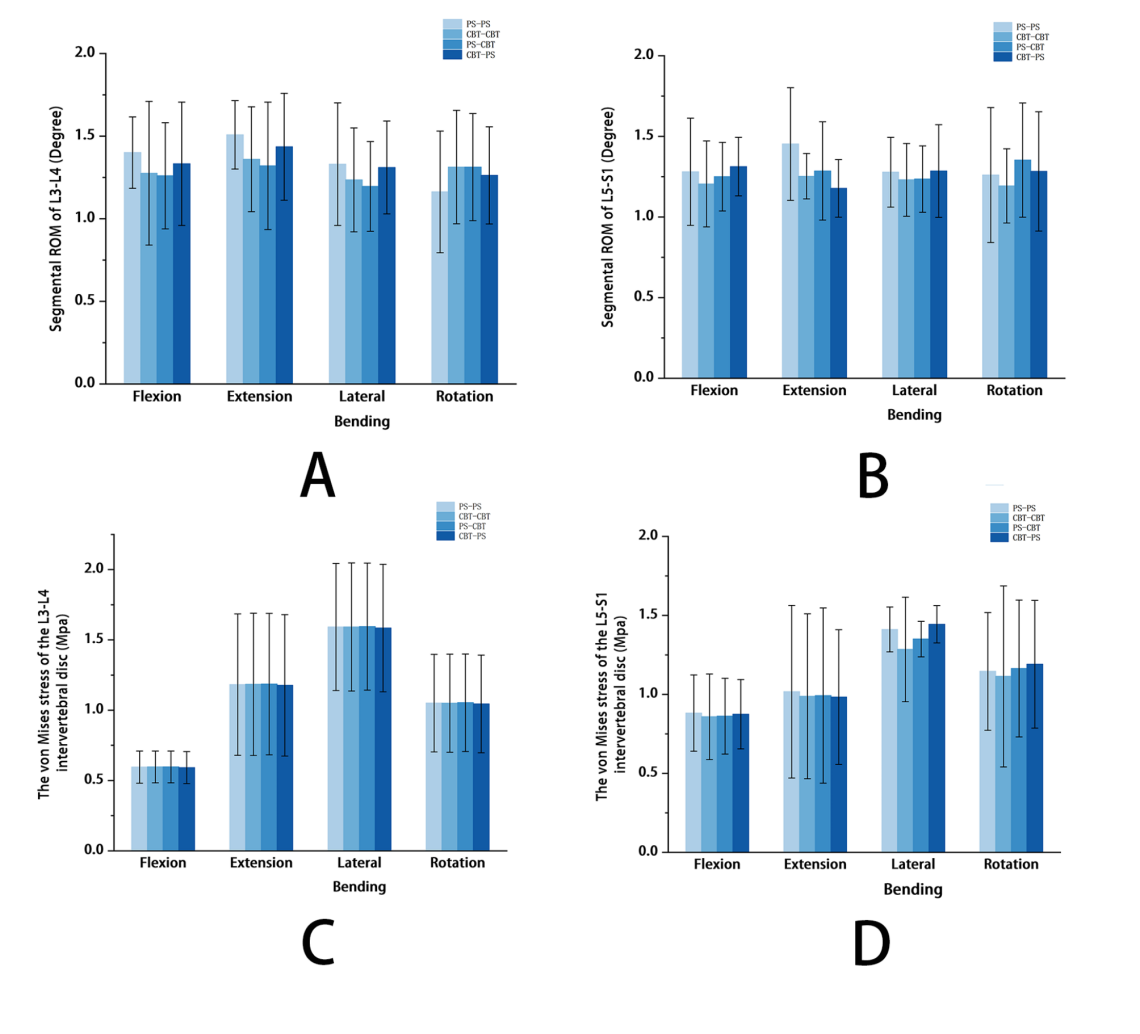
Different biomechanical results of four fixation models. **(A)** ROM of L3-L4 segment; **(B)** ROM of L5-S1 segment; **(C)** von Mises stress of the intervertebral disc at L3-L4 segment; **(D)** von Mises stress of the intervertebral disc at L5-S1 segment.

CBT-PS increased by 5.8%, 8.7% (P = 0.036), and 1.5% in flexion, back extension, and lateral bending compared to PS-CBT and decreased by 8.6% in rotation. The difference between CBT-PS with PS-CBT in back extension was significant (P = 0.036). The ROM of L3-L4 segment for the four types of internal fixation was shown in Fig. 5A.

### ROM of L5-S1 segment

PS-CBT decreased by 2.3%, 11.6%, and 3.4% (P = 0.011) in flexion, extension, and lateral bending, and increased by 7.4% in rotation compared to PS-PS. The difference between PS-PS with PS-CBT in lateral bending was significant (P = 0.011). The ROM of L5-S1 segment for the four types of internal fixation was shown in Fig. 5B.

### Von Mises stress of the intervertebral disc at L3-L4 segment

**Fig. 6:**
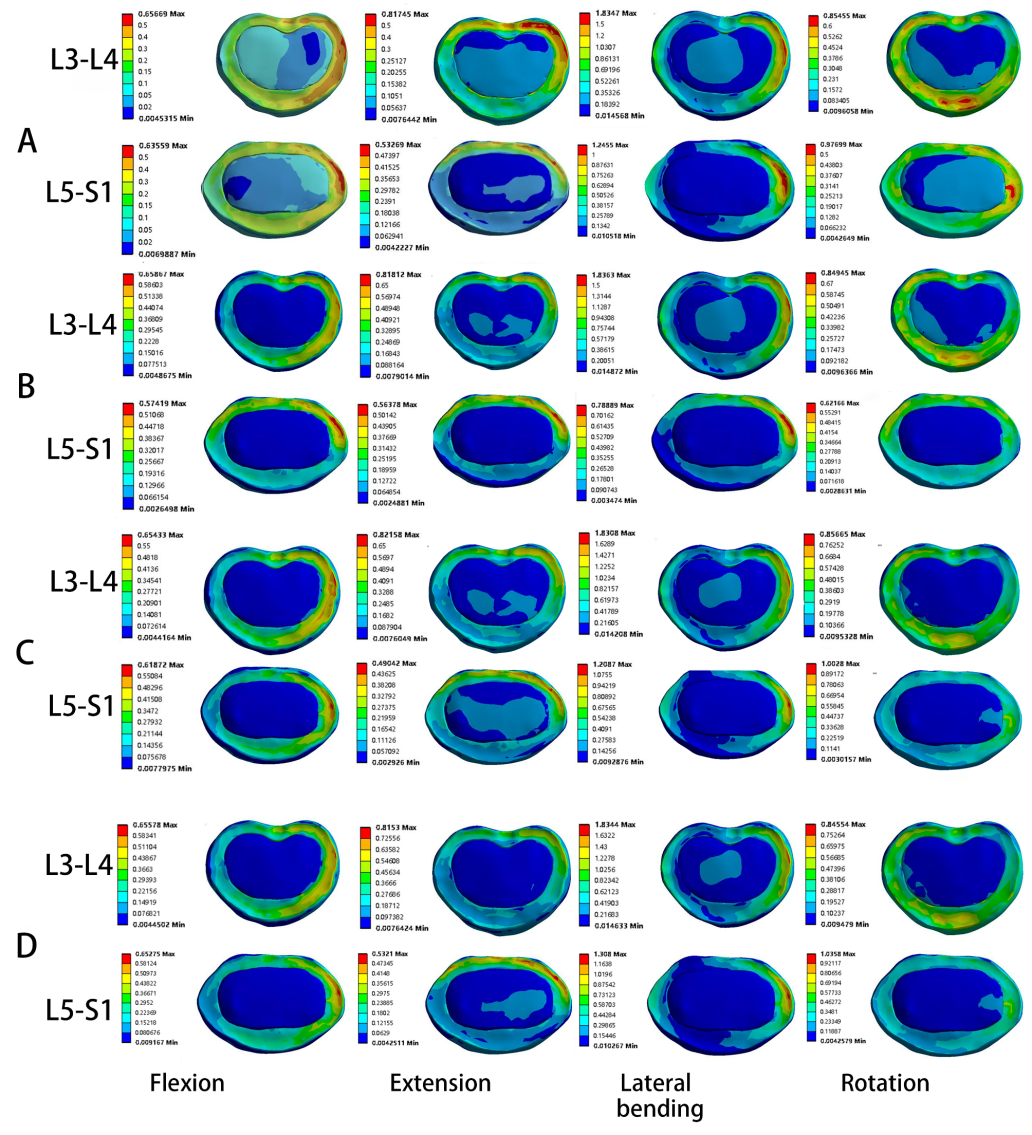
Stress nephograms over the screw in four different fixation models. **(A)** PS-PS; **(B)** CBT-CBT; **(C)** PS-CBT; **(D)** CBT-PS. The screw tail closure area in the PS-PS internal fixation model was defined as “one” and compared with the screw tail closure area in the other three groups of internal fixation models. The PS-CBT model had the largest screw tail area, while the CBT-PS model had the smallest screw tail area.

CBT-PS decreased by 0.54%, 0.37% (P = 0.041), 0.51%, and 0.57% (P = 0.023) in four motions compared with PS-PS. There were some significant differences between CBT-PS and PS-PS in extension (P = 0.041) and axial rotation (P = 0.023). The von Mises stress of the intervertebral disc at L3-L4 segment for the four types of internal fixation was shown in Fig. 5 C. PS-PS showed the most concentrated stress distribution (Fig. 6A), while the CBT-CBT, PS-CBT, and CBT-PS showed a superior load-sharing ability (Fig. 6B, C, D).

### Von Mises stress of the intervertebral disc at L5-S1 segment

PS-CBT decreased by 2.18% (P = 0.006), 2.38% (P = 0.022), and 4.31% in forwarding flexion, posterior extension, and lateral bending, and increased by 2.78% in rotation compared with PS-PS. Compared with PS-CBT, CBT-PS increased by 1.41%, 6.95% (P = 0.046), and 2.25% in flexion, lateral bending, and rotation, and decreased by 0.98% in extension. There was a significant difference between the PS-CBT and PS-PS in forwarding flexion (P = 0.006), and posterior extension (P = 0.022). There was a significant difference between the CBT-PS and PS-CBT in lateral bending (P = 0.046). The von Mises stress of the intervertebral disc at L5-S1 segment for the four types of internal fixation was shown in Fig. 5D. PS-PS showed the most concentrated stress distribution in all motions (Fig. 6A). CBT-CBT, PS-CBT, and CBT-PS showed superior load-sharing abilities (Fig. 6B, C, D).

## Discussion

The occurrence of postoperative ASD was considered to be caused by increased compensation due to the changes in the normal biomechanical environment of the adjacent segments, intraoperative injury to ligaments [29, 30], and the inability to withstand the additional increased stress. In a case study by Lee et al. [4], it was demonstrated that the cranial adjacent segment was more impacted than the caudal adjacent segment. Shono et al. [1] confirmed that the cranial adjacent segment will have a more compensatory role after spinal fusion due to the shift of the spinal motion center cranially. In the axial alignment, the inclination of L1-L2 facet joints presents almost vertically, while L5-S1 facet joints increase in width, shallow in curvature, and deviate coronally towards the facet to prevent the stress of the lumbar spine moving in the frontal direction, and the orientation of the facet joints from upper to lower change gradually from sagittal to coronal position [31]. The upper lumbar spine was weak in the capsule ligaments, with attachment only to the edge of the facet joint (1–2 mm medially). Capsule ligaments further strengthened in the lumbosacral region (13 mm medially). The anatomy of the lower lumbar spine was stronger than that of the upper lumbar spine, so the stability of the lower lumbar spine was superior to that of the upper lumbar spine. Therefore, how to balance the postoperative stress in the adjacent segments requires careful consideration for surgeons. To address these issues, this study further analyzes the capability of two different hybrid fixation techniques in balancing the stress in the adjacent segments. Spinal fusion restricts the motion of the fused segment, increases the stability of the spine, and avoids postoperative failure. However, the implantation of internal fixation results in a redistribution of ROM of each segment. The ROM of the fused segment decreases and compensate by the adjacent segment, resulting in increased ROM of the adjacent segment accelerating the degeneration [1, 4]. Currently, the majority of scholars were using cadaveric specimens for biomechanical studies of the lumbar spine [32, 33], and the subjective factors of the surgeons may affect the experimental results. The FEM can avoid this influence and ensure the accuracy and repeatability of the experiments with idealized screw insertion points and tracts.

The ROM of the adjacent segments (except for L3-L4 segment in rotation) in the present study was greater in PS-PS than in CBT-CBT. Zhang et al. [34] and Matsukawa et al. [18] noted that the ROM of the adjacent segments in CBT technique was greater than in the PS. The results of the present study were not entirely consistent with those of Zhang et al. [34] and Matsukawa et al. [18]. We considered the reasons for the differences were related to the following points: ① The sizes of CBT (diameter of 5.0 mm and length of 35 mm) and PS (diameter of 6.0 mm and length of 45 mm) in this study were different with CBT (diameter in 4.5 mm and length in 30 mm) and PS (diameter in 6.5 mm and length in 45 mm) in Zhang et al. [34]. The diameters of CBT and PS used in this study were decreased, but the length of PS was increased. Karami et al. [32] concluded from in vitro experiment that thicker and longer screws improve the stability of fixation. The length and diameter of CBT in this study were larger than those of Zhang et al. [34]. The length of PS was similar to that of Zhang et al. [34] and the diameter was smaller. Based on the results of Karami et al. [32], we concluded that the stability of CBT-CBT in this study was superior than that of Zhang et al. [34], while the stability of PS-PS was inferior than that of Zhang et al. [34]. This may be one of the reasons for the difference in the ROM of the adjacent segment (except for L3-L4 segment in rotation) in the two studies. ② Differences in the models. In this study, four L1-S1 models were established and facet joint cartilages were defined as “soft frictionless contact” with an initial gap of 0.5 mm, while Zhang et al. [34] established one L3-S1 model and surface contact with the coefficient of friction of 0.1 was used to model the facet joint, and some of the material properties were set with a variation. The surgical approach was different. PLIF was performed in the study of Zhang et al. [34], while TLIF was performed in this study. The TLIF used in this study unilaterally removed the caudal articular process of L4 and the cranial articular process of L5 during the procedure, whereas the PLIF was performed in the study of Zhang et al. [34], it can be seen that the facet joint and surrounding ligamentous structures were not damaged compared to the TLIF.

Wangsawatwong et al. [35] demonstrated that the ROM of PS-PS at the cranial adjacent segment was greater than that of CBT-CBT in flexion and extension, the ROM of the caudal adjacent segment was greater than that of CBT-CBT in lateral flexion and these were partially consistent with the results in this study. This may be due to the following points: ① The sizes of CBT (diameter of 5.0 mm and length of 35 mm) and PS (diameter of 6.0 mm and length of 45 mm) in this study were different from CBT (diameter in 4.5 mm and length ranging from 25 to 35 mm) and PS (diameter in 6.5 mm and length ranging from 45 to 55 mm) in Wangsawatwong et al. [35]. ② The FEM was applied in this study, while in vitro experiment was applied in Wangsawatwong et al. [35]. The hybrid PS-CBT decreased the ROM of L3-L4 segment in four motions, the ROM of L5-S1 segment was lower than PS-PS and greater than CBT-CBT in flexion, extension, and lateral bending, and the maximum was found in rotation. The ROM of L3-L4 segment in CBT-PS was greater than CBT-CBT and lower than PS-PS in flexion, extension, and lateral bending, and greater than PS-PS and lower than CBT-CBT in rotation. The ROM of L5-S1 segment was greater than PS-PS and CBT-CBT in flexion, lateral bending, and rotation. As a result, although CBT-PS has a limited ability to decrease the ROM at the cranial adjacent segment and limit the upward movement of the motion center, it did not produce an extremely higher ROM in one specific motion. In addition, compared to PS-CBT, the adjacent segmental ROM of CBT-PS in flexion, extension, and lateral bending (except for L5-S1 in extension) was greater, and lower in rotation. This indicates that the performance of CBT-PS in decreasing ROM of adjacent segments was inferior to that of PS-CBT, but it provides significant advantages in terms of throughout decompression of nerve and minimizing surgical incision and damage to the facet joint and paravertebral structures in the TLIF procedure.

Stress concentration in adjacent discs will increase the incidence of disc degeneration [33]. Zhang et al. [34] demonstrated that the peak von Mises stress of the disc at the L3-L4 segment was almost identical in PS-PS and CBT-CBT, while the peak von Mises stress of L5-S1 segment of PS-PS was slightly lower than that of CBT-CBT. In this study, the peak von Mises stress of the disc at L3-L4 segment was almost identical in PS-PS and CBT-CBT, whereas it was greater in the PS-PS than CBT-CBT at L5-S1 segment. We concluded the difference was the same as the reasons for the difference in ROM of the adjacent segments.

However, the disc stresses in the cranial segment of PS-PS in the study conducted by Liu et al. [36] were greater than those of CBT-CBT in flexion, extension, and lateral bending, which were somewhat different from the results of the current study. In addition, the distribution of disc stresses at the cranial segment of PS-PS and CBT-CBT were same. The reasons for this difference were as follows: ① The sizes of CBT (diameter of 5.0 mm and length of 35 mm) and PS (diameter of 6.0 mm and length of 45 mm) in this study were different from CBT (diameter in 3.5 mm) and PS (diameter in 5.5 mm) in Liu et al. [36]. ② TLIF with internal fixation technique was performed in this study, while only internal fixation without fusion was performed in the study conducted by Liu et al. [36]. As shown in Fig. 6, the disc stresses during flexion were concentrated in left annulus. This might be associated with the cadaveric specimen being too dated and having some imbalance in the coronal plane. Regarding the disc stresses distribution, among the four different fixation techniques, the disc stresses were most concentrated in both L3-L4 and L5-S1 segments in PS-PS (Fig. 6A), and which was similar in the remaining three fixation techniques. As shown in the disc stresses nephograms, CBT-PS dispersed the disc stresses more effectively (Fig. 6D). The disc stresses at L3-L4 segment showed the maximum in the PS-PS, while CBT-PS showed the minimum.

PS technique requires extensive exposure of the facet joint and the screw insertion point was located around the superior articular process, which causes damage to the articular process. Lee et al. [37] and Marengo et al. [38] compared the clinical efficacy and safety of CBT and PS techniques and found that CBT provided better preservation for the facet joint. TLIF unilaterally removed the caudal articular process of L4 and the cranial articular process of L5. The damage to the facet joint caused by screw occurs in three main ways: ① Initial damage during screw insertion: The insertion point of CBT was located in the vertebral isthmus, away from the facet joint, but the insertion point of PS was located around the facet joint. ② Secondary damage caused by postoperative screw loosening: CBT was more stable than PS in terms of pull-out strength, it may decreases the postoperative screw loosening. ③Damage caused by the rod: In the hybrid fixation techniques with different screw insertion points at L4 and L5, rod need to be fixed across L4-L5 facet joint, and damages the facet joint at L4-L5 segment, but L3-L4 segment.

During the TLIF procedure, decompression of the lateral recess of the caudal lumbar spine was often required to provide throughout decompression of the compressed nerve root. The insertion point of CBT is close to the decompression range and easily affects the decompression effect. The insertion point of PS was located around the facet joint, which was away from the decompression range. In theory, the neurological decompression effect can be achieved with PS at L5 is more significant than that with CBT, so CBT-PS can provide a superior neurological decompression effect.

The study has some limitations. First, the sample size was insufficient. Second, there was no comprehensive analysis of the effect of different sizes of screws on the experimental results. Third, the factors that affect the incidence of ASD were not only the choice of fixation techniques and surgical procedure but also the patient’s specific factors, such as age, BMI, severity of osteoporosis, etc. [1, 3]. Forth, the results of this study need to be further investigated by in vitro study.

## Conclusion

Compared with the PS-PS and CBT-CBT techniques, the PS-CBT and CBT-PS techniques have certain advantages in reducing the impact on the adjacent segments. Although CBT-PS technique was slightly weaker than PS-CBT technique in decreasing the ROM of the cranial adjacent segment, it was capable of achieving an even effect in all motions, distributing the stress concentration at the adjacent segment, decompressing the lateral cress thoroughly, reducing the surgical exposure and damage to the paravertebral tissues as well as decreasing the damage to the facet joint. In addition, CBT-PS technique limits the upward movement of the spinal center allowing the caudal adjacent segment which was more stable to undertake more compensatory effects and avoiding the premature development of ASD.

## Data Availability

The datasets used and/or analysed during the current study are available from the corresponding author on reasonable request.

## List of Abbreviations

TLIF: Transforaminal lumbar interbody fusion
PLIF: Posterior lumbar interbody fusion
ASD: Adjacent segment degeneration
ASDD: Adjacent segment disk degeneration
ROM: Range of motion
CBT: Cortical bone trajectory screw
PS: Pedicle screw
ALL: Anterior longitudinal ligament
PLL: Posterior longitudinal ligament
ITL: Intertransverse ligament
LF: Ligamentum flavum
CL: Capsular ligament
ISL: Interspinous ligament
SLL: Supraspinous ligament
LSD: Least significant difference
