# Correction: Biomechanical evaluation of the hybrid pedicle screw—cortical bone trajectory technique in transforaminal lumbar interbody fusion to adjacent segment degeneration—finite element analysis

**DOI:** 10.1186/s12891-023-06646-w

**Published:** 2023-07-05

**Authors:** Rui Zhang, Alafate Kahaer, Hanqian Niu, Jingwen Wang, Ayididaer Jumahan, Yanning Qiu, Paerhati Rexiti, Hailong Guo

**Affiliations:** 1 Second Clinical Medical College, Xinjiang Medical University, Urumqi, China; 2 Department of Spine Surgery, The First Affiliated Hospital of Xinjiang Medical University, 137 Liyushan South Road, Urumqi, China; 3 Fifth Clinical Medical College, Xinjiang Medical University, Urumqi, China; 4 First Clinical Medical College, Xinjiang Medical University, Urumqi, China; 5 Xinjiang Key Laboratory of High Incidence Disease Research, Xinjiang Medical University, Ministry of Education, Urumqi, China; 6 Xinjiang Orthopedic Clinical Research Center, Urumqi, China

**Correction:** ***BMC Musculoskelet Disord*** **24, 409 (2023)**

10.1186/s12891-023-06411-z

Following publication of the original article [1], we have been notified of two corrections to affiliation changes of the Paerhati Rexiti and authorship changes (order of corresponding authors).

The author group and affiliations have been updated above; and the original article [1] has been corrected.
